# Highway to *heal*: Influence of altered extracellular matrix on infiltrating immune cells during acute and chronic lung diseases

**DOI:** 10.3389/fphar.2022.995051

**Published:** 2022-11-03

**Authors:** Mugdha M. Joglekar, Mehmet Nizamoglu, YiWen Fan, Sai Sneha Priya Nemani, Markus Weckmann, Simon D. Pouwels, Irene H. Heijink, Barbro N. Melgert, Janesh Pillay, Janette K. Burgess

**Affiliations:** ^1^ University of Groningen, University Medical Center Groningen, Department of Pathology and Medical Biology, Groningen, Netherlands; ^2^ University of Groningen, University Medical Center Groningen, Groningen Research Institute for Asthma and COPD (GRIAC), Groningen, Netherlands; ^3^ Department of Paediatric Pneumology &Allergology, University Children’s Hospital, Schleswig-Holstein, Campus Lübeck, Germany; ^4^ Epigenetics of Chronic Lung Disease, Priority Research Area Chronic Lung Diseases; Leibniz Lung Research Center Borstel; Airway Research Center North (ARCN), Member of the German Center for Lung Research (DZL), Germany; ^5^ University of Groningen, University Medical Center Groningen, Department of Pulmonology, Groningen, Netherlands; ^6^ University of Groningen, Department of Molecular Pharmacology, Groningen Research Institute for Pharmacy, Groningen, Netherlands; ^7^ University of Groningen, University Medical Center Groningen, Department of Critical Care, Groningen, Netherlands; ^8^ University of Groningen, University Medical Center Groningen, W.J. Kolff Institute for Biomedical Engineering and Materials Science-FB41, Groningen, Netherlands

**Keywords:** extracellular matrix, migration, infiltrating immune cells, *in vitro* models, lung diseases, three-dimensional

## Abstract

Environmental insults including respiratory infections, in combination with genetic predisposition, may lead to lung diseases such as chronic obstructive pulmonary disease, lung fibrosis, asthma, and acute respiratory distress syndrome. Common characteristics of these diseases are infiltration and activation of inflammatory cells and abnormal extracellular matrix (ECM) turnover, leading to tissue damage and impairments in lung function. The ECM provides three-dimensional (3D) architectural support to the lung and crucial biochemical and biophysical cues to the cells, directing cellular processes. As immune cells travel to reach any site of injury, they encounter the composition and various mechanical features of the ECM. Emerging evidence demonstrates the crucial role played by the local environment in recruiting immune cells and their function in lung diseases. Moreover, recent developments in the field have elucidated considerable differences in responses of immune cells in two-dimensional versus 3D modeling systems. Examining the effect of individual parameters of the ECM to study their effect independently and collectively in a 3D microenvironment will help in better understanding disease pathobiology. In this article, we discuss the importance of investigating cellular migration and recent advances in this field. Moreover, we summarize changes in the ECM in lung diseases and the potential impacts on infiltrating immune cell migration in these diseases. There has been compelling progress in this field that encourages further developments, such as advanced *in vitro* 3D modeling using native ECM-based models, patient-derived materials, and bioprinting. We conclude with an overview of these state-of-the-art methodologies, followed by a discussion on developing novel and innovative models and the practical challenges envisaged in implementing and utilizing these systems.

## 1. Introduction

### 1.1 ECM as the highway for infiltrating immune cells

Cellular migration has a fundamental role in directing development, tissue homeostasis, and disease progression (Morales et al., 2021; Yamada et al., 2022). Cells have different modes of migration–singular, amoeboid or mesenchymal, or collective fashion depending on the local tissue microenvironment and activated signaling pathways (van Helvert et al., 2018; Yamada and Sixt, 2019). The extracellular matrix (ECM) of the lung is a dynamic structural network which consists of proteins, glycosaminoglycans, and glycoproteins (Burgstaller et al., 2017). It provides structural support during important mechanical events of breathing. It is also an important bioactive component of the cellular microenvironment as it provides cues that regulate cellular processes (Theocharis et al., 2016; Yamada and Sixt, 2019). Local molecular composition (including growth factors and cytokines) and biomechanical properties (elasticity, stiffness, and compression forces) of the ECM can govern migration of (infiltrating) immune cells (van Helvert et al., 2018; Yamada and Sixt, 2019; Morales et al., 2021). Other factors that influence cellular migration include confinement of cells, ECM crosslinking and remodeling, and ECM geometry such as topology, fiber alignment, and porosity (van Helvert et al., 2018; Yamada and Sixt, 2019; Morales et al., 2021; Burgess and Harmsen, 2022). Further, ECM fragments resultant from remodeling can promote or inhibit cellular migration (Gu et al., 2018; Nissen et al., 2018; Sharma et al., 2018; de Castro Bras and Frangogiannis, 2020).

The lung is a unique organ exposed to exogenous environmental insults and infectious agents and consequently has highly regulated immune and damage repair responses. Severe or repetitive insults can cause micro-injuries leading to acute and chronic lung diseases (Labaki and Han, 2020). Chronic lung diseases are in general incurable and often have high hospitalization rates. Additionally, some patients are at risk of disease exacerbations that accelerate disease progression. Moreover, insight into the pathobiology of each of the lung diseases is still limited (Labaki and Han, 2020). Therefore, understanding the immunopathology of each of these diseases is essential for developing effective clinical management and new treatment approaches. Chronic obstructive pulmonary disease (COPD), lung fibrosis, asthma, and acute respiratory distress syndrome (ARDS) are all characterized by abnormal ECM turnover and chronic inflammatory responses in varying degrees, which lead to tissue damage (Ito et al., 2019; Burgess and Harmsen, 2022).

Investigating cell-ECM interactions as a contributing factor to the disease progression has been emerging in the last decade (McMahon et al., 2021; Burgess and Harmsen, 2022). Upon injury, the process of tissue repair is initiated, during which recruited immune cells migrate through ECM to reach the target location. Inflammation and resolution of wound healing processes are regulated by contribution of (infiltrating) immune cells (Volk et al., 2013; Manon-Jensen et al., 2016; Wang et al., 2022). It is likely that their migration, in these lung diseases, through aberrant ECM will affect the function of the infiltrating immune cells.

Conventional immune cell migration studies using standard two-dimensional (2D) cell assessment systems have provided conceptual advances (Puttur et al., 2019). Such studies have revealed a specialized mode of migration (repetitive protrusion, adhesion and contraction) that cells adopt in 2D microenvironments (Hallmann et al., 2015; Yamada and Sixt, 2019). However, it is now clear that cells implement several different modes of migration in three-dimensional (3D) environments (Hallmann et al., 2015; Yamada and Sixt, 2019). In both 2D and 3D *in vitro* migration assays the role of ECM in regulating these processes has been explored. Several different materials have been used as ECM-mimicking substrates. These include synthetic polymers such as poly (ethylene glycol) (Widener et al., 2021) and natural polymers such as collagen (Li et al., 2018; Surendran et al., 2021). Methodologies including precision cut lung slices (PCLS), organoids, lung-on-chip, whole decellularized lung tissues, and hydrogels have been developed over the past decades to mimic physiological environments *in vitro*, each with their own advantages and challenges (Gkatzis et al., 2018; Liu et al., 2019; Nizamoglu et al., 2022). However, there has been limited implementation of such models for studying immune cell infiltration, thereby providing future opportunities for exploring the dynamics between ECM and infiltrating immune cell migration in the context of lung diseases.

### 1.2 Flyovers: New discoveries in cellular migration and implications of the ECM highway

As the field moves ahead with innovative models, it is simultaneously important to consider new discoveries in cellular migration and how the inclusion of ECM could add to this knowledge. Cells produce and leave behind retraction fibers during migration that support the formation of vesicle-like structures called migrasomes (Tavano and Heisenberg, 2019; Fan et al., 2022). Cancer cells more frequently migrated along residual retraction fibers in microfluidic channels compared to channels without these fibers (Lee et al., 2021). Neutrophils have been shown to leave cytoplasmic trails containing chemokines for T cells upon viral infection, which may very well consist of migrasomes (Lim et al., 2015). The involvement of the ECM was highlighted through the discovery that the generation of these extracellular vesicles was being triggered by the interaction of cells with fibronectin fibers (Wu et al., 2017; Lee et al., 2021). In concert, migratory trajectories of chemotaxing neutrophils have previously been shown dependent on collagen concentration (Francois et al., 2021). Thus, migrasomes in combination with trails, could increase efficiency of directional migration. The effect of healthy and diseased ECM on the cellular source of migrasomes and trails could begin to explain the continual recruitment of immune cells. Whether a diseased ECM highway provides additional road bumps in the formation of migrasomes and migratory trails remains unexplored. Altogether, these studies highlight the role of the ECM in not just the regulation of the migratory behavior of first responders, but also the recruitment of subsequent immune cells or secondary responses such as adaptive immunity. A study using *Drosophila* embryos demonstrated weakened cell-ECM connections during cellular division that facilitated macrophage infiltration (Akhmanova et al., 2022). Although it may sound counter intuitive, the (increased rate of) division of cells might act as an “exit” from the ECM highway. A similar phenomenon occurring in diseases associated with hyperproliferation of stromal cells such as asthma and fibrotic lung diseases may be possible but it is unexplored to date. These new insights mentioned above on how infiltrating immune cells interact with ECM and the (resident or recruited) cells can also be further expanded in the context of the influence of ECM using *in vitro* models.

In this review, we highlight the importance of interactions between the “highway” ECM and infiltrating cells in the pathogenesis of various lung diseases. We review emerging technologies for *in vitro* modeling that better represent physiological characteristics. Some challenges that exist for implementing these models to study ECM-immune cell interactions during their migration into lung tissue will also be discussed.

## 2. A hazardous highway: Altered ECM in lung diseases and effects on infiltrating immune cells

Knowledge of how ECM relates to cellular migration has been the focus of recent studies illustrating that the ECM acts as a highway for the migrating/infiltrating immune cells. Biochemical and biomechanical properties of ECM influence the migratory behavior of cells, including immune cells. The importance of available adhesion ligands was established when fibroblasts were able to migrate along stiffness gradients (durotaxis) on fibronectin-coated substrates, whereas this ability was lost on substrates coated with laminin (Hartman et al., 2017). Increased fiber alignment promoted cell migration and directionality of migration (Wang et al., 2018). The inability of aged fibroblasts to produce a hyaluronan and proteoglycan cross-linking proteinresulted in the formation of a more aligned matrix that promoted metastasis while inhibiting T cell migration (Kaur et al., 2019). Accelerated ageing is a distinctive feature of some chronic lung diseases such as COPD (Brandsma et al., 2017) and idiopathic pulmonary fibrosis (IPF) (Chilosi et al., 2013; Selman and Pardo, 2021), making the above observation relevant to the field of lung research. In addition to being a reservoir for growth factors and cytokines, other factors of the ECM discussed here collectively influence the migratory behavior of infiltrating immune cells. As the composition of the ECM has been the main focus of many studies so far, most knowledge is on the influence of different ECM components on cell migration. A summary of the changes in composition of lung ECM during lung diseases can be found in Table 1. It is evident from this table that different studies have different conclusions. The diversity in these observations could be attributed to disease heterogeneity, variation in study population, and disease phenotypes. Nevertheless, the table can serve as a guide while developing *in vitro* models within the realms of current knowledge. A simple assumption would be that altered composition of ECM triggers changes in the mesenchymal mode of migration due to the alterations in the number of binding domains available for integrins (Yamada and Sixt, 2019). The following subsections will discuss how infiltrating immune cells participate in lung diseases and what is the role of ECM in influencing these migration patterns in the context of lung fibrosis, COPD, asthma, and ARDS.

**TABLE 1:** Changes in the components of extracellular matrix in lung diseases compared to non-diseased controls (unless otherwise specified). *

ECM proteins	Lung fibrosis	COPD	Asthma	ARDS
Collagen Type I	a) Higher in airways Liu et al. (2021a);b) Higher in fibroblastic foci Herrera et al. (2019)	Lower in inner layer of large and small airways and outer layer of small airways Hogg et al. (2009); Annoni et al. (2012)	a) Higher in small airways Dolhnikoff et al. (2009);b) Higher deposits in reticular basement membrane Hough et al. (2020)	Higher in early and late phase Santos et al. (2006)
Collagen Type III	a) Higher in airways Liu et al. (2021a); b) Higher in fibroblastic foci Herrera et al. (2019)	Relatively higher compared to collagen type I Hogg et al. (2009)	a) Lower in small airways of fatal asthma Dolhnikoff et al. (2009); b) Higher in the airway mucosa Araujo et al. (2008)	Higher in the early phase Santos et al. (2006)
Collagen Type IV	Higher in fibroblastic foci Herrera et al. (2018)	Higher in large airways with epithelial damage Dekkers et al. (2021)	Collagen type IV, alpha 3 deposition is lower in the asthmatic airways Burgess et al. (2010)	Unknown
Collagen Type VI	Higher in fibroblastic foci Herrera et al. (2019)	Higher in airways Abdillahi et al. (2015)	Higher in the alveolar parenchyma of uncontrolled asthmatics Andersson et al. (2018)	Unknown
Other collagens	Collagen type V: higher in fibroblastic foci Herrera et al. (2019)	a) Overall deposition higher in collagen in alveolar walls and small airways walls Eurlings et al. (2014);b) Lower total collagen Hogg et al. (2009)	Collagen type V: higher in airways Liu et al. (2021b)	Unknown
Elastin	Higher in fibrotic areas Burgess et al. (2016)	Lower in alveolar and small airways walls Eurlings et al. (2014)	a) Lower in subepithelium Reddel et al. (2012); b) Higher in submucosa Reddel et al. (2012)	a) Degraded in the early phase Santos et al. (2006);b) Deposited in the late phase Santos et al. (2006)
Fibronectin	Higher in fibroblastic foci Herrera et al. (2019)	Higher in inner and outer layer of small airways, no difference in parenchyma Annoni et al. (2012)	Higher in the outer area of the small airways Dolhnikoff et al. (2009)	Higher in both early and late phase of ARDS Morales et al. (2011); Ito et al. (2019)
Laminin	Unknown	β2: higher in large airways with epithelial damage Dekkers et al. (2021)	ɑ2,3,5 chains: higher epithelial basement membrane expression; ɑ4,5 chains: lower in ASM BM expression Dekkers et al. (2021)	Unknown
Glycosaminoglycans, glycoproteins and proteoglycans	Hyaluronic acid: higher expression in IPF lungs Herrera et al. (2019); Versican: higher expression in fibroblastic foci Herrera et al. (2019); Tenascin-C: higher expression in fibroblastic foci Burgess et al. (2016)	Hyaluronic acid: higher in alveolar and small airway walls Eurlings et al. (2014); Versican: lower in distal parenchyma Annoni et al. (2012); Decorin, biglycan, and lumican: No differences Annoni et al. (2012), lower in peribronchiolar area in severe emphysema van Straaten et al. (1999); Tenascin-C: higher in subepithelial area of large airways and inner layer of small airways Annoni et al. (2012)	Hyaluronic acid: higher in peri- bronchioles and perivascular regions in the lung Lauer et al. (2015);Decorin, lumican, and versican: higher in the subepithelial layer of the airway wall in atopic asthmatics Hough et al. (2020); Biglycan, versican and decorin: higher percentage areas in both central airways and alveolar parenchyma of non-controlled asthma Weitoft et al. (2014)	Versican: higher in small airway walls of patients with fatal ARDS Morales et al. (2011), higher in thickened alveolar walls Bensadoun et al. (1996)

*only the most frequently investigated ECM components are included. Soluble ECM fragments reported in fluids including bronchoalveolar lavage and sputum are beyond the scope of this table.

### 2.1 Lung fibrosis

ECM in lung parenchyma during lung fibrosis is substantially altered from healthy lungs. This has been illustrated both in terms of amounts and/or ratios of ECM components and with respect to the 3D organization of the ECM network (Burgess et al., 2016; Burgstaller et al., 2017; Burgess and Harmsen, 2022; Nizamoglu and Burgess, 2022). Along with altered biochemical composition (Table 1), altered mechanical environment with increased stiffness, decreased viscoelastic relaxation, as well as disorganized fibers and abnormal topography are well-documented changes in ECM in lung fibrosis (Booth et al., 2012; Tjin et al., 2017; de Hilster et al., 2020).

The involvement of circulating immune cells in lung fibrosis is well recognized: among these cells are monocytes and neutrophils (Ishikawa et al., 2021). While the details of recruitment and involvement of these cells are outside scope of this review, these processes can take place through both soluble mediators (Huang et al., 2020; van Geffen et al., 2021) and mechanical factors (Du et al., 2022). Higher counts of monocytes in blood were associated with faster disease progression in interstitial lung diseases (Kim et al., 2022). In mice, monocytes arriving in fibrotic lung tissue transform to macrophages to repopulate lung tissue and remain in the tissue with higher profibrotic activity compared to tissue-resident macrophages (Misharin et al., 2017). Monocytes and neutrophils were found in higher numbers in bronchoalveolar lavage (BAL) fluid of IPF patients (Kinder et al., 2008). Neutrophils were also increased during acute exacerbations of lung fibrosis (Lee et al., 2012). Due to their dynamic nature, both monocytes/macrophages and neutrophils are readily instructed by their microenvironment (Nissen et al., 2018; Vasse et al., 2018; Vasse et al., 2021).

The altered (fibrotic) microenvironment influences infiltrating immune cells in several different ways. In a study, fibroblasts cultured for different durations resulted in varying degrees of fiber organization in collagen matrices (Pakshir et al., 2019). These alterations in fiber organization, however, were unable to influence macrophage migration speed in 3D (Pakshir et al., 2019). On the other hand, neutrophil migration speed but not the directionality, was lower in denser 3D collagen networks (Francois et al., 2021). When fiber crosslinking was applied, increased crosslinking of 2D fibrin surfaces promoted macrophage migration. However, the fiber crosslinking also changed other mechanical parameters, such as stiffness. This unintended change might have also influenced the migrational behavior of the infiltrating immune cells (Hsieh et al., 2019). The influence of ECM crosslinking on migration of neutrophils has yet to be described. Similarly, the influence of altered stress relaxation, another important property of altered microenvironment in fibrotic lung ECM, on infiltrating immune cells has not been examined. New insights for lung fibrosis research can be drawn from a recent study illustrating minimal migration of cancer cells on 2D surfaces which lack stress relaxation, but robust migration of the same cells on the surfaces with high stress relaxation capacity (Adebowale et al., 2021). In addition to the changes in the ECM organization, released ECM fragments can also alter the migratory behavior of the lung resident cells (Nizamoglu and Burgess, 2022). Although there are recent studies focusing on these fragments (Burgess and Harmsen, 2022), their potential influence on migratory behavior and function of infiltrating immune cells remains unknown.

### 2.2 Chronic obstructive pulmonary disease

COPD is characterized by excessive ECM remodeling and ECM deposition around the small airways, while the alveolar region is characterized by ECM disruption and tissue destruction (Burgess et al., 2016; Brandsma et al., 2020). Inflammatory responses are central to COPD and understanding the immunopathology is particularly important as current treatments are ineffective in mitigating disease progression and lung tissue damage. In the context of migration in COPD, neutrophils, monocytes, and T cells to an extent, and have received most attention. These cells, and associated secreted factors, have been reported elevated in patients’ sputum, blood, and BAL, and often correlate with the progression of COPD (Hogg et al., 2004; Vargas-Rojas et al., 2011). In addition, neutrophils and macrophages from patients with COPD display impaired effector functions such as efferocytosis and phagocytosis (Taylor et al., 2010; Tan et al., 2017; Dicker et al., 2018; Belchamber et al., 2019), likely extending to a variation in normal migratory behavior of infiltrating immune cells. This has been previously demonstrated with respect to chemotactic cytokines (Sapey et al., 2011; Costa et al., 2016).

While studies exploring the influence of the ECM and ECM fragments on immune cell migration in COPD are limited, sputum has often been investigated as a chemotactic agent. CD14^+^ monocytes from healthy individuals not only migrated more than CD14^+^ monocytes from patients with COPD, but also more towards COPD sputum compared to normal sputum (Ravi et al., 2017). Similarly, neutrophils from patients with COPD migrated more towards COPD sputum compared to normal sputum, although T cells from these patients did not show the same trend (Wu et al., 2015). These studies did not identify specific sputum factors responsible for the induction of immune migratory responses. Thus, there can be multiple constituents of the sputum that can have chemotactic effects on cells including ECM fragments (Nissen et al., 2018). Indeed, alterations in sputum composition between health and disease have been demonstrated (Titz et al., 2015; Moon et al., 2018), also with respect to differential levels of ECM fragments that can alter cellular migration in patients with COPD. For example, fragments of production or degradation of collagen (Schumann et al., 2018), elastin (Ronnow et al., 2019), and fibrinogen (Manon-Jensen et al., 2019) have also been detected in sputum and serum/plasma in patients with COPD and are proposed as biomarkers of disease progression. Proline-glycine-proline (PGP), a matrikine derived from collagen, is elevated in sputum of patients with COPD and is a potent chemoattractant for neutrophils (Gaggar et al., 2008; O'Reilly et al., 2013; Patel et al., 2018). However, the role of abnormal ECM in recruitment and regulation of migratory behavior of immune cells remains unexplored.

Secreted pro-inflammatory factors such as cytokines and proteases perpetuate immune responses and remodel ECM (Ni and Dong, 2018; Brightling and Greening, 2019). In COPD, higher neutrophil elastase activity was associated with emphysematous tissue destruction (Walton et al., 2016), and lower trans-endothelial T cell migration (Rao et al., 2004). Consequently, biomechanical properties of lung tissue of patients with COPD are altered, such as loss of elasticity, increased stiffness around small airways, and decreased stiffness in the emphysematous regions (Burgess and Harmsen, 2022). These changes are bound to alter the characteristics of cellular migration.

### 2.3 Asthma

Asthma is characterized by hallmark features such as airway inflammation and remodelling. Airway remodeling, a feature of asthma—but also seen in COPD, refers to the structural and ECM changes in both small and large airways (Hough et al., 2020). The profile of ECM is altered in the asthmatic airways with less deposition of collagen type IV, elastin, and more deposition of collagen type I, fibronectin, laminin, periostin, versican, decorin, and lumican (Burgess et al., 2010; Hough et al., 2020; Dekkers et al., 2021). Recently, also fibrillar collagen was shown to be fragmented and disorganized in the lamina propria of large and small airways from patients with asthma (Mostaco-Guidolin et al., 2019). Several factors have been identified in asthma that led to abnormal turnover of ECM components such as epigenetic modifications, recurrent viral infections and excess fibrolysis (Pech et al., 2018; Nemani et al., 2021; Weckmann et al., 2021; Ronnow et al., 2022).

Several immune cells, including neutrophils, eosinophils, monocytes, macrophages, and mast cells, among others, are considered to play an important role in airway remodeling in asthma (Holgate et al., 2015; Helfrich et al., 2019). Alveolar macrophages, mast cells, eosinophils, and neutrophils were shown to degrade ECM by releasing matrix metalloproteinase (MMP)−9 (Hough et al., 2020). MMP-driven degradation of collagen released biologically active fragments in asthma such as the pro-neutrophilic matrikine PGP (Patel and Snelgrove, 2018). Another significant matrikine in asthma is tumstatin, a non-collagenous domain of collagen type IV α3 which was shown to be significantly reduced in airways from patients with asthma (Burgess et al., 2010). Interestingly, when mice were treated with tumstatin the inflammatory cell counts in the lungs were reduced (Burgess et al., 2010).

It was recently suggested that migration of tissue eosinophils in ECM - likely occurs *via* periostin interactions which were particularly higher in T2-high asthmaand correlated with recruitment of eosinophils to the airway (Johansson, 2017; Burgess et al., 2020). Another study reported the chemotaxis of neutrophils was reduced on tumstatin-induced asthmatic airway smooth muscle cell-derived ECM (Harkness et al., 2017). Using a human airway-on-chip, transmigration of immune cells to the epithelial lumen from the vascular microchannel during a viral infection was analyzed. Greatest neutrophil adhesion at the surface of the microvascular endothelium was observed in presence of IL-13 stimulation (to mimic T-helper cell type 2 asthmatic phenotype) rapidly followed by neutrophil trans-endothelial migration through a combination of migratory events (Nawroth et al., 2020).

### 2.4 Acute respiratory distress syndrome

In ARDS, changes in pulmonary ECM are a direct consequence of the inflammatory injury and subsequent repair responses (Tomashefski, 2000; Ito et al., 2019). The changes in ECM can be divided in distinct ARDS phases, with ECM destruction and alveolar and capillary damage predominating in the early phase, which transitions to a fibroproliferative repair phase later. However, the phases are not strictly separated as early fibroblast activation and matrix deposition are also present (Meduri et al., 1998; Boyd et al., 2020).

Early recruitment of neutrophils and monocytes, following lung injury results in ECM degradation, predominantly through the production of MMPs (Torii et al., 1997; Davey et al., 2011). Fragments generated from degradation of ECM play a role in amplifying recruitment of inflammatory cells. The matrikines PGP and its acetylated form induce neutrophil chemotactic activity and migration (Biesalski, 2007; Pierpaoli et al., 2011; Sava et al., 2015; Misiura and Miltyk, 2019; Palmieri et al., 2019). This effect was dose dependent (van Houwelingen et al., 2008) and occurred through C-X-C motif chemokine receptor two interaction on leukocytes (Weathington et al., 2006; Braber et al., 2011; Kim et al., 2011; Hahn et al., 2015; Sharma et al., 2018; Robison et al., 2021). Additionally, the matricellular protein cellular communication network factor 1 (CCN1) was found in high concentrations in BAL fluid of patients with ARDS, while mice overexpressing CCN1 spontaneously developed ARDS coinciding with neutrophil influx (Grazioli et al., 2015; Morrell et al., 2020). The direct effect of CCN1 on cell migration is not straightforward, as it appeared to increase chemokinesis by interaction with αMβ2 integrins. However, prolonged presence of CCN1 inhibited cell migration and played a role in neutrophil clearance through efferocytosis (Lobel et al., 2012; Jun et al., 2015). Fibronectin deposition in the acute phase of ARDS facilitated neutrophil migration partly by higher expression and redistribution of intracellular adhesion molecule-1 (ICAM-1) in endothelial cells (Sava et al., 2015). In a model of *S. aureus* induced skin infection, hyaluronic acid deposition was increased in ARDS and the failure to digest this ECM increased neutrophil influx (Hällgren et al., 1989; Dokoshi et al., 2020).

Less is known about the exact ECM changes in the fibroproliferative phase of ARDS. Synthesis of collagen types I and III was present, but unlike lung fibrosis; at this stage no information on the crosslinking states of these collagens exists. Interestingly, commonalities including similar ECM composition and distribution between IPF and ARDS have been recognized (Raghu et al., 1985). In patients with ARDS, epithelial lining fluid levels of C-terminal propeptide (marker of collagen type I synthesis) were increased, while degradation products of collagen type I/II were reduced compared to individuals at risk of ARDS/ALI (Armstrong et al., 1999). BAL measurement of N-terminal peptide of alveolar procollagen type III, a precursor of collagen type III, has been validated as a diagnostic tool to indicate fibroproliferation in ARDS patients as well as to identify patients who can benefit from corticosteroid treatment (Forel et al., 2015; Hamon et al., 2019). Serological and BAL levels of hyaluronic acid were found associated with ARDS severity and organ failure (Esposito et al., 2017). Recently, lung tissue obtained from patients with coronavirus disease (COVID-19) induced ARDS, stained positively for hyaluronic acid which was associated with the degree of alveolar damage (Hellman et al., 2020). An *in vitro* chemotaxis model recently showed that collagen type III had an inhibitory effect on neutrophil migration regarding track length, direction, and targeting (Kraus et al., 2021). However, it is still unknown whether these mechanisms are active in ARDS.

## 3 *In vitro* modeling

**FIGURE 1:**
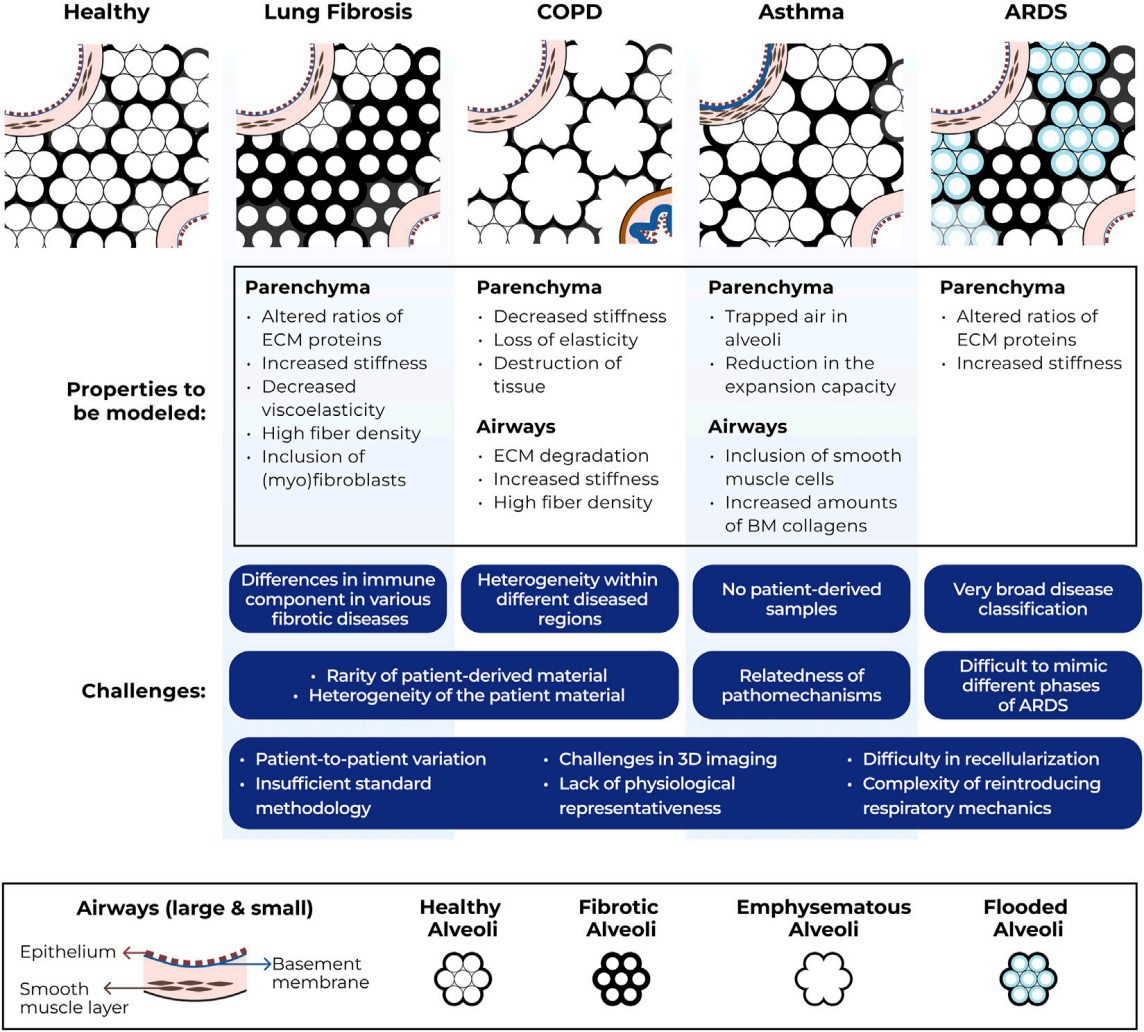
Schematic representation of structural ECM changes in lung diseases, ideal properties for modeling immune cell migration through ECM in these disease conditions and challenges associated with generating such models. COPD = chronic obstructive pulmonary disease, ARDS = acute respiratory distress syndrome, ECM = extracellular matrix, BM = basement membrane, 3D = three-dimensional.

To further strengthen understanding of interactions between infiltrating immune cells and ECM in the context of migration, development of innovative *in vitro* models is key. Some of the ideal properties for *in vitro* modeling of immune cell migration in different types of lung diseases are illustrated in Figure 1.

Moving towards *in vitro* models for studying ECM influences on immune cell migration, ECM-derived *in vitro* models are emerging as a novel methodology. ECM-derived systems have been established using single proteins or by decellularization of native lung tissue. These models recapitulate the biochemical and mechanical properties of native ECM more closely than 2D models in which cells are cultured on plastic with unrepresentative polarity. To facilitate the investigation of altered biomechanics separately or in combination with altered composition of lung ECM, development of novel methodologies and ECM-mimicking biomaterials is warranted. This includes but is not limited to: changing pore size or fiber density without changing ECM-composition or altering mechanical properties without changing fiber density. In a recent study from our group, we demonstrated the possibility of modulating stromal mechanical properties without altering composition (Nizamoglu et al., 2022). Another study implemented macromolecular crowding to induce changes in the collagen fibril networks, without significantly changing the bulk stiffness (Ranamukhaarachchi et al., 2019).

In addition to native ECM-based models, patient-derived materials are an important source of cells that are essential for establishing *in vivo* representative models. Involving multiple cell types (such as epithelial cells or fibroblasts) adds to the physiological relevance of a model and these cell-cell interactions can provide invaluable information about disease-driving mechanisms. Effects of cell-cell interactions on immune cell migration has been demonstrated for many types of immune cells, such as between peripheral blood-derived monocytes and leukocytes (Costa et al., 2016), between fibroblasts and macrophages (Ford et al., 2019), and between epithelial spheroids and neutrophils (Surendran et al., 2021). Investigating the influence of these cell-cell interactions on (infiltrating) cell migration within the context of diseased ECM could bring new perspectives to our current understanding of lung disease pathobiology.

### 3.1 Challenges associated with 3D migration models

Patient-derived material has the highest physiological relevance when used for modeling *in vitro* systems, however, such samples pose various challenges associated with their nature. The availability of human lung tissue for scientific research is rare, except in some specialized clinical centers. Moreover, large volumes of tissue cannot be obtained for every disease; for instance, for asthma and ARDS usually only small bronchial biopsies are available. Furthermore, obtaining true healthy control tissue is an added obstacle. Control “healthy” material is often obtained from lung tissue resected during lobectomies, tumors, or transplantation. The resected tissue is assessed for morphological and anatomical normalcy and although the cells and tissues may appear to be healthy, their microenvironment is possibly altered as a consequence of disease compared to a healthy individual. Patient-to-patient variability creates additional challenges while working with the small(er) sample sizes that are inherent to such models. Modeling chronic diseases should also be accompanied by modeling with appropriate controls, which include important considerations such as matching for age, sex, and smoking history. However, the limitations in the availability of precious patient material also constrains the inclusion of proper controls to perform appropriate comparisons. This challenge of limited availability of donor material also extends to models that utilize human-derived ECM. Another important consideration for ECM-based models is heterogeneity of mechanical properties in different compartments of the available human material. For example, small airways in COPD become stiffer but the parenchyma on the whole appears softer due to enlarged emphysematous regions while the remaining alveolar walls are measured as having stiffness similar to control alveolar walls (Burgess and Harmsen, 2022). There are several well-established protocols to decellularize the lung to obtain either intact scaffolds or solubilized ECM, that is, reconstituted to form hydrogels (Wagner et al., 2013; Gilpin and Wagner, 2018; de Hilster et al., 2020). An unmet challenge for these models, however, is recellularization (Wagner et al., 2013). Current efforts at recellularization are unable to ensure appropriate 3D distribution of cells. Advances in 3D bioprinting technology such as ECM based bioinks reinforced with cells can bolster the development of models with correct spatial distribution of cells (De Santis et al., 2021; Falcones et al., 2021).

Conducting experiments in 3D provides a plethora of information in addition to the physiological relevance. An extra dimension goes hand-in-hand with added challenges for retrieving readouts to generate these data. Visualizing the network of ECM with varying degrees of resolution, is possible using histological staining (Masson Trichrome, Picrosirius Red), immunohistochemistry and/or immunofluorescence staining, scanning electron microscopy, and atomic force microscopy. However, sample processing techniques may limit the extent of visualization and/or introduce artefacts. For example, sectioning the sample for staining procedures limits the information provided to only one plane, the harsh treatments necessary for scanning electron microscopy sample preparation can alter ECM structure. Similarly, fluorescence imaging approaches might be hindered by auto-fluorescence of native ECM. Second harmonics generation and multiphoton microscopy are emerging as prominent high-resolution imaging techniques for visualizing the matrix and overcoming these limitations (Mayorca-Guiliani et al., 2017; Tjin et al., 2017). Fluorescent-labeling of cells or matrix has also allowed deciphering matrix changes and cellular movement in different studies (Fischer et al., 2022). Digital holographic microscopy has been utilized to visualize cell migration in 3D Matrigel matrices (Hellesvik et al., 2020). While each visualization method has advantages and disadvantages (Martinez-Garcia et al., 2022), combination of different techniques for the visualization of migrating cells and the ECM network might be the key for advancing knowledge.

Another important feature of the lung is the presence of oxygen gradient. The alveolar-arterial (A-a) oxygen gradient has been previously used as an indicator of disease severity and outcome in pneumonia and recently in COVID-19 (Singh et al., 2022). Often, acute and chronic lung diseases are also characterized by hypoxemia and hypoxia. Thus, modeling gradients *in vitro* systems, although challenging, is crucial as cells modulate their responses depending on the oxygen levels in their microenvironment (Zenewicz, 2017). Most 3D migration systems are modeled under static conditions, missing the dynamic state of the lung. Inclusion of respiratory mechanics associated with breathing and blood flow also poses a challenge while modeling these systems. One such event is cyclic deformations which have been mimicked in lung-on-chip models recently (Kumar et al., 2022; Zhu et al., 2022). Including cyclic deformations in the state-of-the-art ECM-based migration models would increase the translational capacity of the models and bring them one step closer to *in vivo*. Similarly, the lack of (interstitial) flow is an important aspect that can add another dimension to these migration models. The effect of interstitial flow was elucidated when tumor-associated interstitial flow promoted tumor-like characteristics in healthy macrophages (Li et al., 2018). Similarly, neutrophils were shown to infiltrate cancer-derived spheroids deeper when a flow was present in the *in vitro* system (Surendran et al., 2021).

Altogether, using innovative 3D *in vitro* models to mimic migration of infiltrating immune cells in lung diseases has been emerging as a new possibility. Developing new systems to represent altered ECM composition, structure, organization and mechanics in each of these lung diseases will help us advance our understanding how the ECM-immune cell interplay influences the migration of these cells.

## 4 Conclusion: Highway to *heal*

Interactions with the microenvironment critically direct cell behavior, including cell migration. Therefore, it is highly likely that disrupted ECM homeostasis in lung diseases such as lung fibrosis, COPD, asthma and/or ARDS alters the behavior of infiltrating inflammatory cells, similar to how a hazardous highway would hinder the smooth flow of traffic. Advances in methodologies for 3D culture systems and advances in the biomaterials field in the last decade have greatly improved our understanding of how migrating cells interact with their microenvironment with respect to the biochemical and biomechanical properties. Emerging data suggest that the contributions of different ECM properties may differ when assessed individually as compared to when in combinations. Targeting isolated parameters within an altered ECM is one of the important questions upon which future research should focus. Another important aspect that remains unknown is the influence that lung-resident cells, such as epithelial cells, endothelial cells and fibroblasts, have on migration of immune cells. Multicellular *in vitro* models are necessary to investigate whether resident lung cells modulate immune cell migration through abnormal ECM in lung diseases. Developing 3D ECM *in vitro* models helps to further our understanding of the pathobiology of a disease (Tabdanov et al., 2021). Recently, modulating cancer ECM has been shown to have potential for therapeutic targeting as weakening cell-matrix adhesion and reducing fiber rigidity reduced cancer cell invasiveness (Pal et al., 2021). Therefore, it is not unlikely that similar approaches targeting the contribution of altered ECM to immune cell recruitment could be employed as therapeutic strategies against lung diseases.

The lack of techniques to obtain information from these novel models poses a future challenge. Nevertheless, steady progress has led to advances in new qualitative and quantitative methodologies for studying disease mechanisms using 3D models. Newer approaches for better imaging, improved compositional analyses, recellularization, and modeling dynamic conditions are paving the way for improved and innovative models. Incorporation of patient-derived material such as native ECM and cells in research will play an important role in our understanding of disease origin and progression.

In summary, understanding the recruitment of immune cells from peripheral blood during lung diseases and how the diseased ECM alters their behavior is a key factor to deepen our knowledge of these diseases and to start generating hypotheses revolving around targeting these interactions for the development of new treatment strategies.
